# Enhanced Immune Functions of In Vitro Human Natural Killer Cells and Splenocytes in Immunosuppressed Mice Supplemented with Mature Silkworm Products

**DOI:** 10.3390/nu17030417

**Published:** 2025-01-23

**Authors:** Thanh Thi Tam Nguyen, Byungki Jang, Seong-Ruyl Kim, Sang-Kuk Kang, Kee-Young Kim, Yoo Hee Kim, Young Ho Koh

**Affiliations:** 1 Department of Biomedical Gerontology, Hallym University Graduate School, Chuncheon 24252, Republic of Korea; tamnguyen@hallym.ac.kr; 2 Ilsong Institute of Life Science, Hallym University, Seoul 07441, Republic of Korea; jang@hallym.ac.kr (B.J.); yooii@hallym.ac.kr (Y.H.K.); 3 Division of Industrial Insects and Sericulture, National Institute of Agricultural Sciences, Wanju 55365, Republic of Korea; ksr319@korea.kr (S.-R.K.); wkdudghl@korea.kr (S.-K.K.); applekky@korea.kr (K.-Y.K.)

**Keywords:** innate immunity, NK cells, immunosuppression, splenocytes, mature silkworm

## Abstract

Objectives: The immune-enhancing properties of steamed mature silkworm, known as HongJam (HJ), were investigated using human interleukin-2-independent Natural Killer 92 (NK92-MI) cells and a cyclophosphamide intraperitoneal injection-induced immunosuppressed mice model (CPA-IP). White Jade variety mature silkworm HJ (WJ-HJ) was used to prepare WJ-HJ supercritical fluid extracts (WJ-SCE) and WJ-HJ-supplemented feeds. Results: Treatment with WJ-SCE significantly enhanced proliferation, migration, and cytotoxicity of NK92-MI cells against various cancer cells while improving mitochondrial function and ATP production (*p* < 0.05). In CPA-IP mice, consumption of WJ-HJ-supplemented feeds restored immune function by improving body weight, immune organ indices, immunoglobulin levels, and blood cytokines. Splenocyte proliferation and cytotoxicity were significantly elevated in both saline intraperitoneal injection (Sal-IP) and CPA-IP groups with WJ-HJ supplementation, independent of mitogen activation (*p* < 0.05). Conclusions: These results suggest that WJ-HJ enhances immune modulation and immune surveillance functions of NK cells by improving mitochondrial and cytotoxic functions. WJ-HJ holds promise as a functional food for immune enhancement, pending clinical validation.

## 1. Introduction

The immune system is essential for maintaining health by defending against external threats and internal abnormalities that disrupt homeostasis [1]. It comprises two main components: innate immunity, which provides the first line of defense through immune cells such as macrophages, natural killer (NK) cells, and dendritic cells, and adaptive immunity, which is mediated by T- and B-lymphocytes activated by the innate system [2]. Dysregulation of the immune system is linked to numerous diseases, including cancer, autoimmune conditions, and neurodegenerative disorders, and can be triggered by factors like infections, aging, medications, or genetic predispositions.

Among innate immune cells, NK cells are central to immune modulation and surveillance, performing cytotoxic and cytokine-mediated roles. They destroy abnormal cells via death receptor activation or by releasing cytolytic granules containing perforin, granzymes, and TRAIL [3,4,5,6,7,8]. NK cells also produce cytokines such as IFN-γ in response to tumor ligands, pathogens, or inflammatory signals, modulating both innate and adaptive immune responses [9,10].

Cyclophosphamide (CPA), an alkylating agent in cancer therapy and autoimmune disease management, is known for its immunosuppressive side effects, including reduced immune organ function, leukopenia, and altered cytokine production [11,12,13,14]. CPA-induced immunosuppressed mice models are therefore widely used to evaluate immune-enhancing or immunomodulating agents.

Silkworms have long been consumed as a sustainable protein source and are traditionally used in Eastern medicine for their health benefits, such as improving metabolic, cognitive, and gastrointestinal functions [15,16,17,18]. The Republic of Korea’s National Institute of Agricultural Sciences (NIAS) recently developed HongJam (HJ), a steamed product made from mature silkworms with high silk protein content [19,20]. Preclinical studies suggest HJ may extend healthspan, improve memory, and support liver and gastrointestinal health [16,21]. However, its potential immune-enhancing properties have not yet been investigated.

This study explores the immune-enhancing effects of HJ, focusing on its impact on NK cell activity and immune modulation using human NK92-MI cells and a CPA-induced immunosuppressed mice model. We hypothesize that HJ enhances immunity by improving mitochondrial function, NK cell cytotoxicity, and overall immune system recovery in CPA-IP-induced immunosuppressed mice models.

## 2. Materials and Methods

### 2.1. HongJam Production and Supercritical Fluid Extraction Protocol

The White Jade silkworm variety (WJ) was raised on mulberry leaves at the National Institute of Agricultural Science (NIAS) campus in Wanju-gun, Jeollabuk-do, Republic of Korea. The WJ-HongJam (WJ-HJ) was produced following previously published protocols [22,23]. Mature silkworms were collected, rinsed with tap water, and steamed for 130 min in a pressure-free cooking unit (Kum Seong Ltd., Bucheon, Kyeonggi-do, Republic of Korea). The WJ-HJ was freeze-dried at −50 °C for 24 h using a freeze-dryer (FDT-8612, Operon Ltd., Kimpo, Kyeonggi-do, Republic of Korea) and ground into powder using a stone roller mill (Duksan Co. Ltd., Siheung, Kyeonggi-do, Republic of Korea). Voucher specimens of WJ-HJ have been deposited in the Silkmoth Quality Maintenance and Storage Laboratory, Division of Industrial Insect and Sericulture, NIAS, Wanju-gun, Jeollabuk-do, Republic of Korea. The nutrient contents of WJ-HJ were previously published [22,23].

A supercritical fluid extractor (Suflux 1 L SC-CO_2_ Extraction System, ILSHIN Autoclave, Daejeon, Republic of Korea) was used to obtain WJ-HJ supercritical fluid extracts (WJ-SCE), following the manufacturer’s guidelines. The system was set up as follows: the reservoir temperature was maintained at 22–25 °C, the extractor at 58–62 °C, the separator at 39–41 °C, and both the primary and secondary heaters at 75 °C, with a CO_2_ flow rate of 0.02–0.04 L/min. The extraction pressure was set at 50–52 bar for the reservoir, 345–355 bar for the extractor, and 49–51 bar for the separator. The extraction process was conducted on 100 g of WJ-HJ powder for 1.5 h. After extraction, the remaining WJ-HJ powder appeared fully dried, as oils and other extractable molecules were removed. Extraction efficiency, calculated by dividing the weight of the extracted solution by the initial weight of WJ-HJ powder, was 12.0%.

### 2.2. Cell Lines, Cell Culture, NK92-MI Cell Viability, Migration, and Cytotoxicity Assay

The interleukin-2-independent human natural killer (NK) cell line, NK92-MI, purchased from the American Type Culture Collection (ATCC, Manassas, VA, USA), was propagated utilizing alpha minimum essential medium (αMEM, Biowest, Nuaillé, France) supplemented with 12.5% horse serum (Thermo Fisher Scientific, Waltham, MA, USA), 12.5% fetal bovine serum (FBS, Biowest), 0.2 mM inositol (Merck KGaA, Darmstadt, Germany), 0.1 mM β-mercaptoethanol (Merck KGaA), and 0.02 mM folic acid (Merck KGaA), along with penicillin (100 U/mL) and streptomycin (100 μg/mL) (antibiotics, Biowest). The K562 and AsPC-1 cell lines (Korean Cell Line bank, Seoul, Republic of Korea) were cultured in RPMI 1640 (Biowest) supplemented with 10% FBS and antibiotics (Biowest). U-373MG (Korean Cell Line bank) were cultured in high-glucose Dulbecco modified Eagle medium (DMEM) (Biowest) supplemented with 10% (*V*/*V*) FBS and antibiotics (Biowest). All cells were cultured under conditions of 37 °C and 5.0% CO_2_ in an incubator.

The cells were treated with 0.1, 0.5, and 1.0 mg/mL of WJ-SCE for 24 h at 37 °C with 5% CO_2_. Cell viability was assessed using the Quanti-Max™ WST-8 cell viability assay kit (BioMax, Guri, Kyeonggi-do, Republic of Korea). A cell suspension of 5 × 10^4^ cells/well was seeded into a 96-well plate and incubated for 24 h before adding different concentrations of WJ-SCE. Following an additional 24 h incubation, WST-8 reagent was added to each well and incubated for 4 more hours at 37 °C with 5% CO_2_. Absorbance was then measured at 450 nm using a BioTek microplate spectrophotometer (Epoch, BioTek, Dover, MA, USA). Six biological replicates were conducted.

To investigate whether WJ-SCE can enhance the migration ability of NK92-MI cells, a previously published protocol [24] was modified. Bottom wells of a 24-well plate were filled with serum-free media, with or without WJ-SCE. Then, 2.5 × 10^5^ NK92-MI cells in 0.2 mL serum-free media were added into a 24-well insert hanging chamber with an 8.0 μm pore size (SPLInsert™, SPL, Pocheon, Kyeonggi-do, Republic of Korea). After an 8 h incubation at 37 °C with 5% CO_2_, cell migration was assessed using the Quanti-Max™ WST-8 cell viability assay Kit (BioMax), quantifying the number of NK92-MI cells that migrated into the bottom wells, as described previously.

To investigate whether WJ-SCE enhances the cytotoxicity of NK92-MI cells against various cancer cells, a lactate dehydrogenase (LDH)-based cytotoxicity assay was conducted using a Quanti-LDH™ PLUS Cytotoxicity Assay Kit (BioMax). NK92-MI cells were pre-incubated with various concentrations of WJ-SCE for 24 h. Target cancer cells (5 × 10^3^ cells per well) were plated in a 96-well plate and incubated for 24 h before adding NK92-MI cells at a 10:1 effector-to-target ratio. After a 6 h co-culture, 100 μL of the supernatant from each well was collected and transferred to a new plate, followed by the addition of 50 μL of a substrate mix. The plates were then incubated at room temperature for 30 min, and absorbance at 490 nm (A490) was measured to evaluate cytotoxicity.

The percentage of NK92-MI cell cytotoxicity was calculated using the following formula:

Cytotoxicity (%) = A − low control/(high control − low control) × 100,(1)

Here, “A” represents the difference in LDH release between the effector–target cell mixture and the effector cell control. The “low control” corresponds to the spontaneous LDH release from the target cells, while the “high control” represents the maximum LDH activity released from the target cells after complete lysis with a cell lysis buffer.

### 2.3. Optical Imaging Protocol of Killing U-373MG Cells by NK92-MI Cells Treated with Various Concentrations of WJ-SCE

To assess the cytotoxic effects of WJ-SCE-treated NK92-MI cells on U-373MG cells via optical imaging, U-373MG cells (1.0 × 10^4^) in 0.5 mL of complete medium were seeded into a 24-well plate and incubated for 36 h. After the medium was removed, 0.3 mL of 0.2 μg/mL Hoechst 33342 (Merck, KGaA) in incomplete medium was added and incubated for 30 min. NK92-MI cells were pre-treated with 0.1, 0.5, or 1.0 mg/mL of WJ-SCE for 24 h. Following three washes with complete medium, 0.3 mL of complete medium was added to the untreated control. NK92-MI cells, either untreated or treated with varying WJ-SCE concentrations, were then prepared for cytotoxicity comparison.

Next, NK92-MI cells (4.0 × 10^4^) in 0.3 mL of complete medium were added to each well and incubated for 4 h. After the medium was completely removed, 0.5 μM of propidium iodide (PI) in Dulbecco’s phosphate-buffered saline (DPBS, Biowest) was added and incubated for 15 min. Fluorescence imaging was performed using a fluorescence microscope equipped with a digital camera (KI-3000F, Korea Lab Tech, Seongnam, Republic of Korea).

For cell identification, U-373MG cells were visualized by blue fluorescence from Hoechst 33342-stained DNA, while dead cells were detected by red fluorescence from PI-stained nuclei. NK92-MI cells were visible in phase-contrast images without blue fluorescence signals.

### 2.4. Total RNA Isolation and Real-Time Quantitative PCR Analysis

After incubation of NK92-MI cells with 0.1, 0.5, and 1.0 mg/mL of WJ-SCE for 48 h, the cells were harvested for total RNA extraction using Trizol reagent, following the manufacturer’s protocol (Thermo Fisher Scientific). The quality of total RNA was assessed by measuring the absorbance at 260 nm/absorbance at 280 nm ratio and the 28S rRNA/18S rRNA ratio. Genomic DNA contaminants were removed using DNase I (Enzynomics, Daejeon, Republic of Korea), and cDNA synthesis was conducted with SuperScript IV (Thermo Fisher Scientific).

The DNA sequences of primers and real-time quantitative PCR (RT-qPCR) conditions are provided in Table S1. RT-qPCR was carried out using SYBR Green PCR Master Mix (Thermo Fisher Scientific) on a QuantStudio 3 Real-Time PCR Instrument (Thermo Fisher Scientific). Each analysis was performed in three biological replicates. The relative gene expression levels were quantified using the 2^−ΔΔCT^ method, as previously published [25]. β-actin was used as a loading control. 

### 2.5. Protocols for Mitochondria Extraction, MitoCom I–IV Activity, and ATP Quantification Assays

Mitochondria complex (MitoCom) I–IV assays were conducted as previously published [15,25]. After incubating NK92-MI cells with various concentrations of WJ-SCE for 24 h, cells were harvested and homogenized in mitochondrial extraction buffer (MEB; 250 mM sucrose, 250 mM mannitol, 2 mM EGTA, 1% (*w*/*v*) BSA, 5 mM Tris-HCl, pH 7.4, Merck KGaA). Mitochondria were isolated by centrifugation at 600× *g* for 5 min at 4 °C and resuspended in ice-cold MEB. After a subsequent centrifugation at 10,000× *g* for 10 min at 4 °C, mitochondrial pellets were dissolved in ice-cold MEB for MitoCom I and II assays or in ice-cold MEB with 1 mM n-D-β-D-maltoside for MitoCom III and IV assays.

For the MitoCom I assay, 20 μg of isolated mitochondrial samples was mixed with the MitoCom I assay buffer (25 mM PB (pH 7.8), 0.35% BSA, 60 μM dichlorophenolindophenol, 70 μM decylubiquinone, 1 μM antimycin A, 0.02 mM NADH, 5 mM NADH). The absorbance at 600 nm (A600) was then measured over 4 min at 37 °C using a Multiskan Go spectrophotometer (Thermo Fisher Scientific). For the MitoCom II assay, 20 μg of isolated mitochondrial samples was prepared with MitoCom II assay buffer (80 mM KH_2_PO_4_ (pH 7.8), 0.1% BSA, 2.0 mM EDTA, 0.2 mM ATP, 80 μM dichlorophenolindophenol, 50 mM decyl-ubiquinone, 1.0 mM antimycin A, and 3.0 mM rotenone). Samples were incubated for 10 min at 37 °C, followed by the addition of 4.0 μL of 0.1 M KCN and 2 μL of 1.0 M succinate. After a 10 min incubation, A600 was measured over 5 min.

The enzyme activity for both MitoCom I and II assays was calculated using the following formula:

Activity = (ΔA600/min × volume assay)/[(extinction coefficient of DCIP × volume of sample) × (protein concentration)](2)

Extinction coefficient of dichlorophenolindophenol at 600 nm: 19.1 mM^−1^.

For MitoCom III activity assays, 20 μg of the mitochondrial sample was mixed with the MitoCom III assay buffer (50 mM Tris-HCl (pH 7.5), 0.1 mM decylubiquinone, 12.5 mM succinate, 2 mM potassium cyanide, 30 μM rotenone, 40 μM cytochrome C, and 4 mM NaN_3_). Absorbance changes at 550 nm (A550) were recorded at 30 °C for 3 min. MitoCom III activities were calculated using the following formula:

Milli OD/min/µg protein = [(ΔA550 sample − ΔA550 blank)/min]/(21.84 × volume of sample × protein concentration)(3)

For MitoCom IV activity measurements, 0.22 mM cytochrome c (Merck KGaA) was mixed with 0.1 M dithiothreitol (Merck KGaA) and incubated for 15 min at room temperature (RT) to ensure complete reduction. A 5 μg mitochondrial sample was then combined with MitoCom IV assay buffer (10 mM Tris-HCl, pH 7.0, 120 mM KCl, and 11.0 μM ferrocytochrome c). A550 was measured 7 times at 10 s intervals at 25 °C. MitoCom IV activity was calculated as follows:

Enzyme activity (µmol/min/mg) = (ΔA550 × volume assay)/[(21.84 × volume of sample) × (protein concentration)],(4)

where 21.84: ΔзmM between ferrocytochrome C and ferricytochrome C at 550 nm.

A previously published ATP assay protocol was used [15,25]. NK92-MI cells treated with various concentrations of WJ-SCE were lysed using a phenol-saturated TE buffer (1:100, Merck KGaA), and then 15% chloroform and 10% dH_2_O (Merck KGaA) were added. After vortexing and centrifuging at 10,000× *g* for 5 min at 4 °C, supernatants were collected for ATP quantification, while the precipitates were retained to determine protein concentrations by a BCA method.

Supernatants or ATP standard solutions were mixed with an ATP assay buffer (50 μM luciferin, 1.25 μg/mL luciferase, 1 mM dithiothreitol, 20.875 mM tricine, 4.175 mM MgSO_4_, 0.835 mM EDTA, and 0.835 mM NaN_3_, Merck KGaA). Luminescence was measured using a Victor Nivo™ multimode plate reader (PerkinElmer, Waltham, MA, USA). ATP levels in samples were calculated using a standard curve and normalized against the protein concentration of each sample.

### 2.6. The CPA-IP-Induced Immunosuppression Mouse Models: Immune Organ Indices, Blood Collecting, and Determination of Plasma Immunoglobulins and Cytokinases

Four-week-old specific pathogen-free C57BL/6J female mice were acquired from Saeron Bio (Uiwang-si, Republic of Korea) and housed at the animal facility at Doheon research center, Hallym University Hangang Sacred Hospital, Seoul, Republic of Korea. Animal experiments were approved (1 November 2022) and supervised by the Experimental Animal Ethics Committee at Hallym University (HMC 2022-0-1014-40). The planning, conducting, and analysis of animal experiments adhered to the guidelines of Animal Research: Reporting In Vivo Experiments [26]. Thirty-two mice were randomly divided into four groups and housed under controlled conditions: 23 ± 1 °C, 50 ± 10% humidity, and a 12/12 h dark/light cycle, with access to normal mouse feed (Nf) or one of three WJ-HJ-supplemented feeds (0.5, 1.0, or 2.0 g/kg body weight (BW)) and water ad libitum. Details of the administered experimental substances are presented in Figure S1.

From days 73 to 77, mice received daily intraperitoneal injections of either saline (Sal-IP) or CPA-IP (80 mg/kg BW). Each group consisted of eight mice, randomly assigned to either the Sal-IP or CPA-IP treatment. IP injections were administered without special measures, as they caused only momentary pain. However, the animals were continuously monitored for abnormalities throughout the study. No sudden weight loss exceeding 20% or abnormal behaviors were observed in any of the animals during the experiment. After a five-day recovery period, all mice were euthanized with a home-made CO_2_ euthanizer, and then blood samples were collected into anticoagulant-treated test tubes. The samples were centrifuged at 3000 rpm for 15 min at 4 °C to separate plasma, which was then used for immunoglobulin and cytokine analysis. Additionally, the weights of the spleen, thymus, and liver were immediately recorded and normalized to BW to calculate immune organ indices.

Immunoglobulin G (IgG), IgA, and IgM levels in the blood of Sal- and CPA-IP mice were measured using enzyme-linked immunosorbent assay (ELISA) with a mouse monoclonal antibody isotyping kit (Merck KGaA). Diluted samples were added to a 96-well plate and incubated at RT for 1 h, followed by three washes with PBST. Next, 100 μL of each isotype-specific monoclonal antibody was added, and the plate was incubated at RT for 30 min before being washed three times with PBST. Subsequently, 100 μL of peroxidase-labeled goat anti-mouse IgG antibody was added, incubated for 15 min at RT, and washed twice. A substrate solution containing 5-aminosalicylic acid and 1% hydrogen peroxide was then added (100 μL), and the plate was incubated for an additional 15 min at RT. The reaction was stopped by adding 50 μL of 3 N NaOH, and absorbance was measured at 450 nm using an Epoch microplate reader (BioTek).

The levels of interleukin (IL)-1β, TNF-α, and IL-10 in plasma were quantified using ELISA kits (Thermo Fisher Scientific) according to the manufacturer’s instructions. Fifty microliters of plasma and fifty microliters of biotin-conjugated antibodies specific to each cytokine were added to a pre-coated 96-well plate and incubated at RT for 2 h, followed by two washes with wash solution. After adding 100 μL of streptavidin–HRP antibodies, the plates were incubated for 1 h at RT and washed again. Then, 100 μL of TMB solution was added, and the plates were incubated at RT for 15 min. Finally, 100 μL of stop solution was added, and absorbance was measured at 450 nm. Cytokine concentrations were calculated using a standard curve.

### 2.7. Preparation for Mouse Ex Vivo Primary Splenocyte Cultures Followed by Splenocyte Proliferation and Cytotoxicity Assays

Spleen tissues from the euthanized mice were harvested, weighed, and immediately placed in RPMI 1640 medium (Biowest). Spleens were mechanically dissociated by being pressed through a 70 μm nylon cell strainer (SPL) using the plunger of a syringe to isolate splenocytes. The resulting cell suspension was centrifuged at 1200 rpm for 5 min to pellet the cells. Erythrocytes were lysed by resuspending the pellet in red blood cell lysis buffer (Merck KGaA) and incubating on ice for 5 min. After another centrifugation at 1200 rpm for 5 min, the splenocytes were washed twice with DPBS. Finally, the cell pellet was resuspended in complete RPMI 1640 medium supplemented with 10% FBS and antibiotics, and cell numbers were counted using a Scepter 3.0 Handheld Automated Cell Counter (Merck KGaA).

The viabilities and proliferations of splenocytes from Sal- and CPA-IP groups were investigated. Primary cultured splenocytes from each group were plated in a 96-well plate at a density of 1 × 10^6^ cells/mL and were either left untreated or treated with concanavalin A (ConA) for T-cell activation or lipopolysaccharide (LPS) for B-cell activation. After 48 h of incubation, a Quanti-Max™ WST-8 cell viability assay kit was used to assess cell viability as described above.

Splenocytes were prepared as effector cells for the LDH-based cytotoxicity assay, with K562 cells serving as target cells. Effector cells (1 × 10^5^ cells/well) were plated in 96-well plates and co-cultured with target cells (2 × 10^3^ cells/well). After 24 h of co-culture, an LDH-based cytotoxicity assay was performed as described above.

### 2.8. Statistical Analysis

Statistical analysis was performed using SPSS Statistics 25 (IBM, Chicago, IL, USA) with Shapiro–Wilk (SW) normality tests and one-way analysis of variance followed by Tukey’s honestly significant difference post hoc analysis. A *p*-value less than 0.05 was considered statistically significant. Different letters above error bars indicate significant differences at *p* < 0.05.

## 3. Results

### 3.1. WJ-SCE Enhanced Proliferation and Migration Abilities of NK92-MI Cells

**Figure 1:**
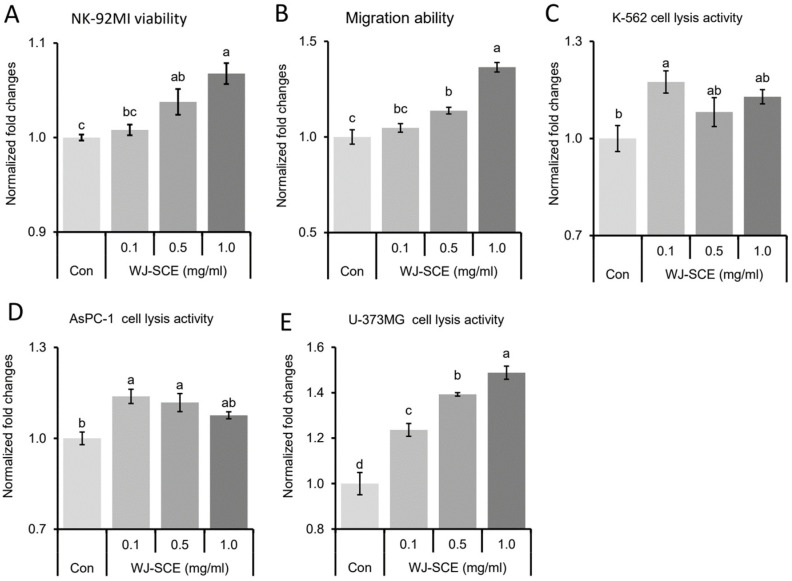
Enhanced proliferation, migration, and cytotoxicity of NK92-MI cells by WJ-SCE. (**A**,**B**) WJ-SCE significantly enhanced NK92-MI cell proliferation (**A**) and migration (**B**) enhancement effects of WJ-SCE. Additionally, WJ-SCE significantly increased the cytotoxicity of NK92-MI cells toward (**C**) K-562; (**D**) AsPC-1; and (**E**) U-373MG cell lines. The letters above error bars indicate significant differences at *p* < 0.05.

To investigate the potential of WJ-SCE in enhancing innate immunity, we first assessed its effects on NK92-MI cell proliferation. Compared to the untreated control (Con) group, WJ-SCE-treated groups exhibited a significant, dose-dependent increase in NK92-MI cell proliferation (Figure 1A, F_(3,23)_ = 12.894, *p* = 6.5 × 10^−5^). A key function of NK cells in innate immunity is to locate pathogen-infected or abnormal cells while circulating through the body. Therefore, we evaluated the migration abilities of NK92-MI cells. Supplementation with more than 0.5 mg/mL of WJ-SCE significantly enhanced the migration of NK92-MI cells (Figure 1B, F_(3,16)_ = 30.913, *p* = 6.8 × 10^−7^). These findings suggested that WJ-SCE may enhance innate immunity by promoting the proliferation and migration of NK cells.

### 3.2. WJ-SCE Enhanced Cytotoxic Activities of NK92-MI Cells Against Cancer Cells

To assess whether WJ-SCE enhances the cytotoxic activity of NK92-MI cells against cancer cells, three different cancer cell lines were tested. When K562, a human lymphoblast cell line, was co-cultured with WJ-SCE-treated NK92-MI cells, a significant increase in cytotoxicity was observed at a concentration of 0.1 mg/mL WJ-SCE (Figure 1C, F_(3,11)_ = 4.21, *p* = 0.046). Similarly, co-culture of AsPC-1, a pancreatic adenocarcinoma cell line, with NK92-MI cells treated with varying concentrations of WJ-SCE showed significantly enhanced cytotoxic activity (Figure 1D, F_(3,11)_ = 12.882, *p* = 0.002). Moreover, when U-373 MG, a human Grade III astrocytoma cell line, was co-cultured with NK92-MI cells, a significant enhancement in cytotoxicity was noted in all WJ-SCE-treated groups (Figure 1E, F_(3,11)_ = 44.518, *p* = 2.5 × 10^−5^). These findings suggest that WJ-SCE not only enhances the proliferation and migration of NK92-MI cells but also boosts their cytotoxicity against various cancer cell types.

**Figure 2:**
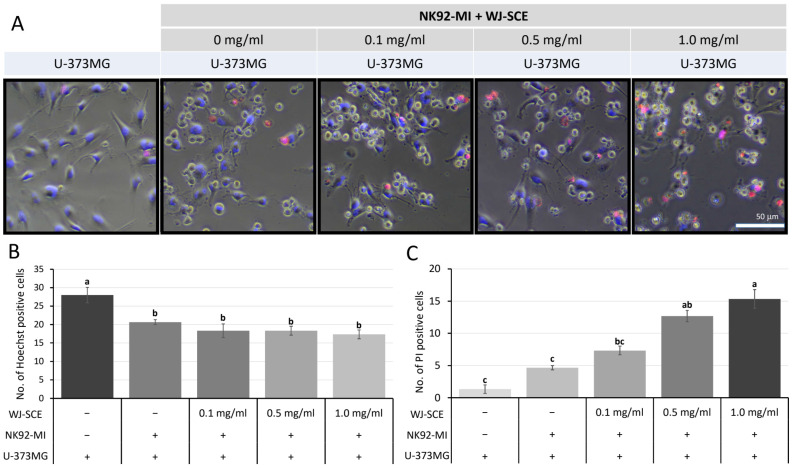
In vivo imaging of U-373MG cells treated with NK92-MI cells. (**A**) U-373MG cells cultured alone or with various NK92-MI cell treatments were sequentially stained with Hoechst 33342 and PI, enabling clear distinction between NK92-MI cells, live U-373MG cells, and dead cells. (**B**) The number of Hoechst 33342-positive (live) cells was significantly reduced in groups treated with NK92-MI cells. (**C**) The number of PI-positive (dead) cells increased in a WJ-SCE dose-dependent manner. The letters above error bars indicate significant differences at *p* < 0.05.

To provide further evidence whether NK92-MI cells could destroy U-373MG cells, we used Hoechst 33342, a genomic DNA staining dye for live cells, and propidium iodide (PI), which stains the nuclei of dead cells. We confirmed that 0.2 µg/mL of Hoechst 33342 was non-toxic to U-373MG cells. Hoechst 33342 bound to the nuclear DNA of all U-373MG cells, displaying blue fluorescence signals, while NK92-MI cells, not stained with Hoechst 33342, appeared as small round cells in phase-contrast images (Figure 2). Dividing U-373MG cells also adopted into a round shape but were clearly distinguishable from NK92-MI cells due to Hoechst 33342 staining. The nuclei of dead U-373MG cells were stained with PI and showed red fluorescence signals. Compared to U-373MG cells not treated with NK92-MI cells, groups treated with NK92-MI cells showed a significant reduction in U-373MG cell numbers (Figure 2B, F_(4,10)_ = 8.551, *p* = 0.003). Furthermore, the number of dead cells indicated by red signals significantly increased in a dose-dependent manner with WJ-SCE treatments (Figure 2C, F_(4,10)_ = 42.17, *p* = 3.1 × 10^−6^). These findings, consistent with the NK92-MI cytotoxicity assay results above, demonstrate that WJ-SCE enhances the cytotoxicity of NK92-MI cells against cancer cells.

### 3.3. WJ-SCE Enhanced Mitochondria Functions and Increased ATP and ROS Amounts in NK92-MI Cells

**Figure 3:**
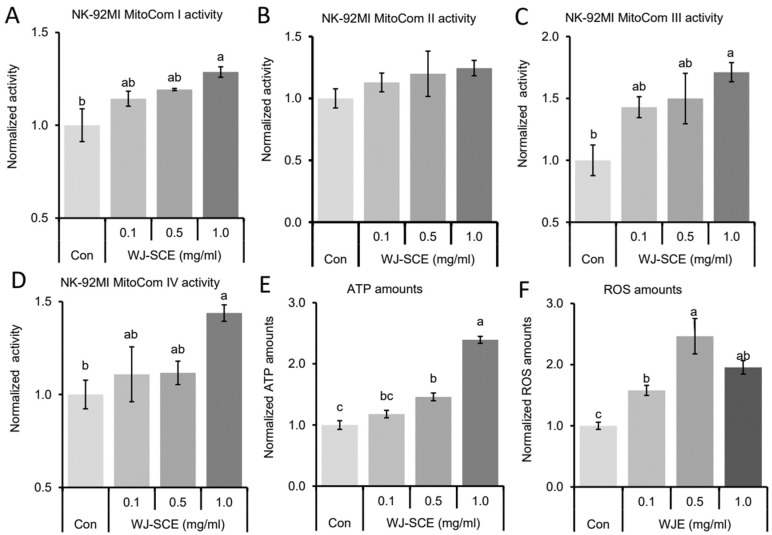
Enhanced MitoCom I–IV activities and increased ATP levels in NK92-MI cells treated with WJ-SCE. (**A**) MitoCom I activity showed significant differences among NK92-MI cells treated with various concentrations of WJ-SCE. (**B**) No significant differences were observed in MitoCom II activity across tested NK92-MI cells. (**C**) MitoCom III activity was significantly enhanced in groups treated with more than 1.0 mg/mL of WJ-SCE. (**D**) Similarly, MitoCom IV activity was significantly increased in the 1.0 mg/mL WJ-SCE-treated group. (**E**) ATP levels showed significant increases in groups treated with 0.5 mg/mL or more of WJ-SCE compared to the Con. (**F**) ROS levels in WJ-SCE-treated NK92-MI cells were significantly higher than in the Con. Different letters above error bars indicate significantly different groups at *p* < 0.05.

In previous studies, we demonstrated that the brains of mice fed with HJ showed significantly enhanced mitochondrial function [15,25,27]. In addition, a recent study reported that NK92-MI anticancer activity was enhanced by supplementing with healthy mitochondria [28]. Therefore, we investigated mitochondrial activity in NK92-MI cells treated with various concentrations of WJ-SCE. Mitochondrial complex I (MitoCom I) activity in WJ-SCE-treated NK92-MI cells was significantly enhanced compared to the Con (Figure 3A, F_(3,11)_ = 5.585, *p* = 0.023). However, MitoCom II activity showed no significant difference in WJ-SCE-treated NK92-MI cells compared to the Con (Figure 3B, F_(3,11)_ = 0.93, *p* = 0.47). In contrast, MitoCom III activity showed a significant, dose-dependent increase in WJ-SCE-treated NK92-MI cells (Figure 3C, F_(3,11)_ = 5.106, *p* = 0.029). MitoCom IV activity also showed a significant increase in the 1.0 mg/mL WJ-SCE treatment (Figure 3D, F_(3,11)_ = 4.257, *p* = 0.045). Furthermore, ATP levels were significantly increased in NK92-MI cells treated with concentrations of WJ-SCE ≥ 0.5 mg/mL (Figure 3E, F_(3,11)_ = 96.214, *p* = 1.3 × 10^−6^). ROS levels were also significantly higher in WJ-SCE-treated groups than in the Con (Figure 3F, F_(3,11)_ = 14.356, *p* = 2.8 × 10^−4^).

These results suggested that WJ-SCE enhanced mitochondrial functions, increasing ATP and ROS levels in NK92-MI cells, which might be a biochemical basis for enhanced functions observed in WJ-SCE-treated NK92-MI cells.

### 3.4. Upregulation of NK Cell Markers, Cytotoxic Enzymes, and Receptors by WJ-SCE

**Figure 4:**
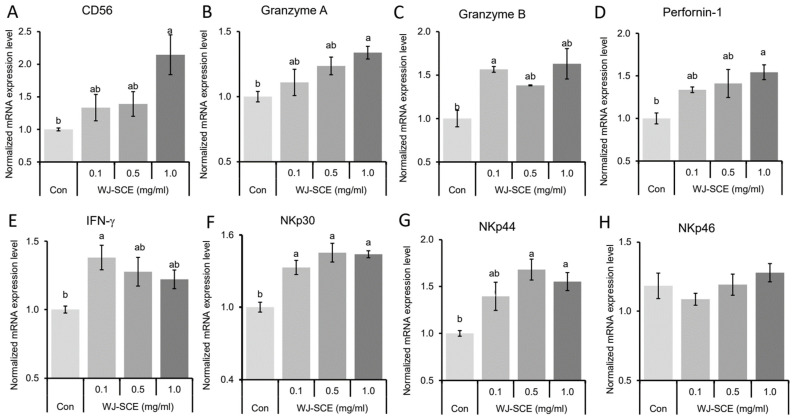
WJ-SCE upregulated expressions of immune-stimulatory genes. The expressions of CD56, Granzyme A, Granzyme B, Perforin-1, INF-γ, NKp30, NKp44, and NKp46 mRNAs were measured by RT-qPCR analysis in NK92-MI cells treated with various concentrations of WJ-SCE. (**A**) Significant differences in CD56 mRNA expressions were observed between the Con and WJ-SCE-treated cells. (**B**) Granzyme A mRNA expression was significantly upregulated in a dose-dependent manner. (**C**) Significant differences in Granzyme B mRNA expressions were detected between the Con and 0.1 mg/mL WJ-SCE-treated NK92-MI cells. (**D**) Perforin-1 mRNA expression was significantly increased in a dose-dependent manner by WJ-SCE treatment. (**E**) IFN-γ mRNA expression showed significant differences between the control and 0.1 mg/mL WJ-SCE-treated cells. (**F**) NKp30 mRNA expression was significantly higher in WJ-SCE-treated cells compared to the Con. (**G**) NKp44 mRNA expression was significantly increased in the 0.5 and 1.0 mg/mL WJ-SCE-treated cells compared to the Con. (**H**) No significant differences were observed in NKp46 mRNA expression among the tested groups. Different letters above error bars indicate significantly different groups at *p* < 0.05.

To investigate the molecular basis underlying the enhancement of proliferation, migration, and cytotoxicity of NK92-MI cells by WJ-SCE, we examined the expression changes of key regulatory or cytotoxic genes. Neural cell adhesion molecule 1 (NCAM/CD56) is a phenotypic marker of NK cells and plays a crucial role in regulating their proliferation and cytotoxicity against tumors or pathogen-infected cells [29]. Compared to the Con group, there was significantly increased CD56 mRNA expression in the 1.0 mg/mL of WJ- SCE treated group (Figure 4A, F_(3,11)_ = 5.483, *p* = 0.024). Granzymes and perforin-1 are major components of cytotoxic granules which NK cells release to destroy target cells [30,31]. The expression of granzyme A mRNA was significantly and dose-dependently increased in WJ-SCE-treated NK92-MI cells (Figure 4B, F_(3,11)_ = 4.601, *p* = 0.037). The expression of granzyme B mRNAs was significantly increased in WJ-SCE-treated NK92-MI cells (Figure 4C, F_(3,11)_ = 7.871, *p* = 0.009). In addition, the expression of perforin-1 mRNA was significantly increased in the 1.0 mg/mL of WJ-SCE-treated NK92-MI cells (Figure 4D, F_(3,11)_ = 5.353, *p* = 0.026). IFN-γ is a proinflammaotry cytokine secreted from NK cells and enhanced their cytotoxicities [32]. The expression of IFN-γ mRNA was significantly enhanced in WJ-SCE-treated NK92-MI cells (Figure 4E, F_(3,11)_ = 14.612, *p* = 0.001). Since the natural cytotoxic triggering receptors such as NKp30, NKp44, and NKp60 regulate NK-mediating tumor cell lysis [33], their mRNA expression changes in NK92-MI cells treated with WJ-SCE were tested. The expressions of NKp30 mRNAs (Figure 4F, F_(3,11)_ = 14.612, *p* = 0.001) and NKp44 mRNAs (Figure 4G, F_(3,11)_ = 7.703, *p* = 0.01) in WJ-SCE-treated NK92-MI cells were significantly higher than those in the Con. However, there was no significant difference in the expression of NKp60 mRNAs among groups (Figure 4H, F_(3,11)_ = 1.12, *p* = 0.371). These gene expression analysis results suggested that WJ-SCE can enhance proliferation and cytotoxicity by promoting expression of NK cell markers, cytotoxic enzymes, and certain receptors.

### 3.5. WJ-HJ Supplementation Recovered CPA-Induced Immunosuppression Phenotypes in Mice

**Figure 5:**
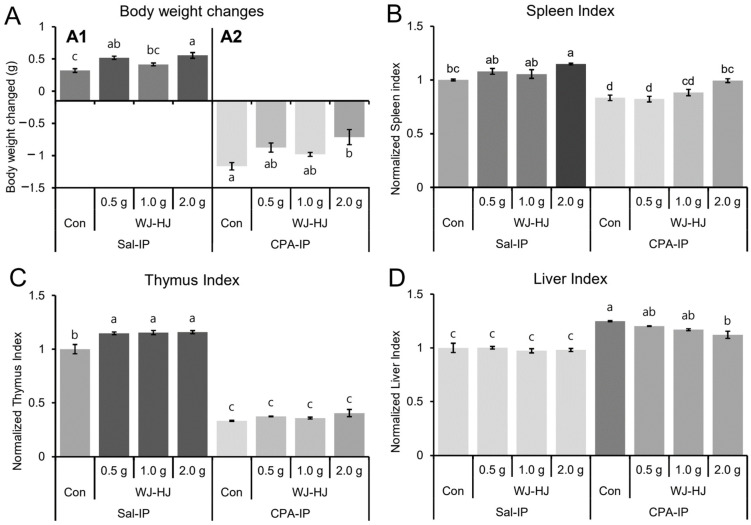
Changes in BWs and immune organ indices in the Sal-IP and CPA-IP groups supplemented with WJ-HJ. (**A**) BW changes in two groups. (**A1**) Significant differences in BW were observed among the Sal-IP group. (**A2**) The reduced BW caused by CPA-IP was significantly restored in the 2.0 g WJ-HJ subgroup. (**B**) Significant differences in spleen index were noted between the Sal-IP and CPA-IP groups. The spleen index was highest in the 2.0 g WJ-HJ subgroup of the Sal-IP group and lowest in the Con subgroup of the CPA-IP group. (**C**) The thymus index also showed significant differences between the Sal-IP and CPA-IP groups, with significantly lower values in the CPA-IP group compared to the Sal-IP group. (**D**) Liver index differences were significant between the Sal-IP and CPA-IP groups. The elevated liver index in the Con subgroup of the CPA-IP group was significantly reduced in the 2.0 g WJ-HJ subgroup. Different letters above or below error bars indicate significant differences at *p* < 0.05.

To assess whether WJ-HJ could enhance immunity in vivo, a CPA-IP model was investigated. CPA-IP models have previously been reported to show reduced BWs and immune organ indices [34,35]. Significant differences in BW were observed among tested groups compared to the Sal-IP group (Figure 5(A1), F_(3,12)_ = 11.649, *p* = 0.0007). The BWs of the 0.5 g and 2.0 g WJ-HJ subgroups within the Sal-IP group were significantly higher than those in the Con subgroup of the Sal-IP group. Additionally, BW reductions were observed in the CPA-IP group compared to the Sal-IP group (Figure 5(A2), F_(3,12)_ = 6.202, *p* = 0.0087), and the 2.0 g WJ-HJ subgroup showed significantly higher BW than the Con subgroup within the CPA-IP group.

When spleen indices of the Sal-IP and CPA-IP groups were compared, significant differences were found among the tested groups (Figure 5B, F_(7,24)_ = 22.852, *p* = 3.7 × 10^−9^). The significantly reduced spleen indices in the Con subgroup of the CPA-IP group were restored in the 1.0 g and 2.0 g WJ-HJ subgroups. Additionally, the spleen indices in the three WJ-HJ subgroups within the Sal-IP group were significantly higher than in the Con subgroup. Regarding thymus indices, the WJ-HJ subgroups in the Sal-IP group exhibited significantly higher thymus indices than the Con subgroup, while significantly reduced thymus indices were noted in the CPA-IP group (Figure 5C, F_(7,24)_ = 334.97, *p* = 2.4 × 10^−22^). For liver indices, there was no significant difference in the Sal-IP group while there were significantly higher liver indices in the CPA-IP group (Figure 5D, F_(7,24)_ = 22.927, *p* = 4.6 × 10^−9^). Notably, the liver index in the 2.0 g WJ-HJ subgroup within the CPA-IP group was significantly lower than in the Con subgroup of the CPA-IP group.

These analyses of BWs and immune organ indices suggested that WJ-HJ supplementation effectively restored CPA-IP-induced immunosuppression phenotypes.

### 3.6. WJ-HJ Supplementation Restored Immunoglobulin and Cytokine Levels Reduced by CPA-IP

**Figure 6:**
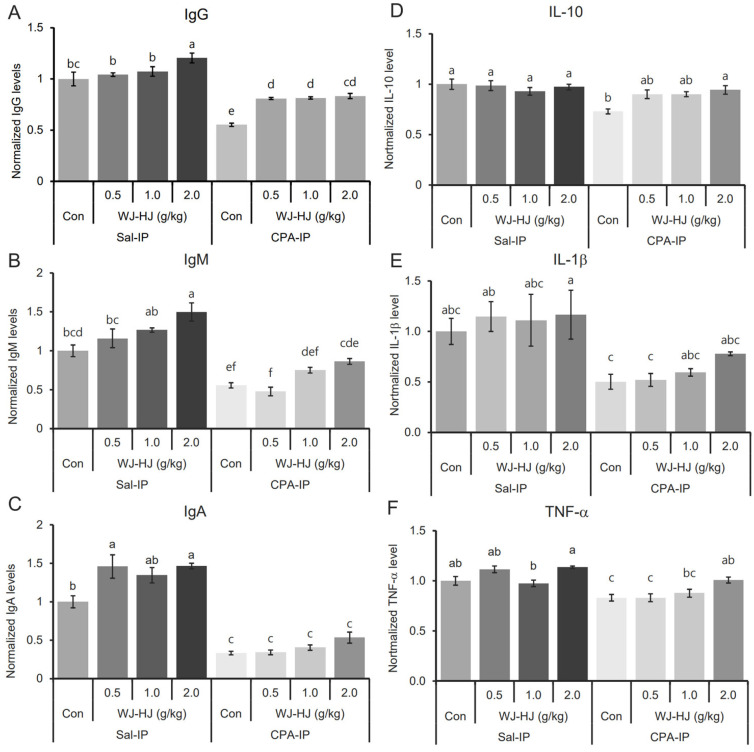
Restoration of Ig and cytokine levels in CPA-IP mouse models through WJ-HJ supplementation. (**A**) Significant differences in blood IgG levels were observed between the Sal-IP and CPA-IP groups. IgG levels were highest in the 2.0 g WJ-HJ subgroup within the Sal-IP group and lowest in the Con subgroup of the CPA-IP group. (**B**). Blood IgM levels showed significant differences across the Sal-IP and CPA-IP groups, with IgM levels reduced by CPA-IP and restored in the 1.0 g and 2.0 g WJ-HJ supplementation subgroups. (**C**) IgA levels also differed significantly between the Sal-IP and CPA-IP groups. The 0.5 g and 2.0 g WJ-HJ subgroups in the Sal-IP group showed significantly higher IgA levels than the Con subgroup, while CPA-IP-induced IgA reduction appeared to be mitigated in the WJ-HJ-supplemented subgroups. (**D**) IL-10 levels were significantly reduced in the Con subgroup of the CPA-IP group compared to the Sal-IP group and the 2.0 g WJ-HJ subgroup in the CPA-IP group. (**E**) Significant differences in IL-1β levels were found among the Sal-IP and CPA-IP groups, with the Con and 0.5 g WJ-HJ subgroups in the CPA-IP group showing lower IL-1β levels than other subgroups. (**F**) TNF-α levels were significantly lower in the Con and 0.5 g WJ-HJ subgroups of the CPA-IP group compared to other subgroups in the Sal-IP and CPA-IP groups. Different letters above error bars indicate significant differences at *p* < 0.05.

Another known phenotype of the CPA-IP model is the reduction of blood Igs and cytokines. Therefore, the levels of various Igs in the blood of the Sal-IP and CPA-IP groups were examined. The CPA-IP group showed significantly reduced Ig levels (Figure 6). Notably, WJ-HJ supplementation in CPA-IP mice restored the reduced IgG levels in the blood. IgG levels in the 2.0 g WJ-HJ subgroup of the Sal-IP group were significantly higher than those in the Con subgroup of the Sal-IP group (Figure 6A, F_(7,24)_ = 32.36, *p* = 9.9 × 10^−11^). Reduced IgM levels in the Con subgroup of the CPA-IP group were restored in the 1.0 g and 2.0 g WJ-HJ subgroups. Additionally, the 2.0 g WJ-HJ subgroup in the Sal-IP group showed significantly higher IgM levels compared to the Con subgroup in the Sal-IP group (Figure 6B, F_(7,24)_ = 24.023, *p* = 2.2 × 10^−9^). The significantly reduced IgA levels in the Con subgroup of the CPA-IP group were also restored in the 2.0 g WJ-HJ subgroup. In the Sal-IP group, the 0.5 g and 2.0 g WJ-HJ subgroups had significantly higher IgA levels than the Con subgroup (Figure 6C, F_(7,24)_ = 43.418, *p* = 4.2 × 10^−12^).

IL-10 levels in the Con subgroup of the CPA-IP group were significantly lower than those in the Sal-IP group and the 2.0 g WJ-HJ subgroup of the CPA-IP group (Figure 6D, F_(7,16)_ = 4.798, *p* = 0.005). IL-1β levels in the Con and 0.5 g WJ-HJ subgroups of the CPA-IP group were significantly lower than those in the 0.5 g and 2.0 g WJ-HJ subgroups in the Sal-IP group (Figure 6E, F_(7,16)_ = 3.807, *p* = 0.013). WJ-HJ supplementation appeared to increase IL-1β levels in the CPA-IP group. Similarly, TNF-α levels in the Con and 0.5 g WJ-HJ subgroups of the CPA-IP group were significantly lower than in other subgroups of both the Sal-IP and CPA-IP groups (Figure 6F, F_(7,16)_ = 12.046, *p* = 2.5 × 10^−5^).

These analyses of blood Igs and cytokines suggested that WJ-HJ supplementation could restore altered blood Ig and cytokine levels in CPA-IP mice by enhancing the activities of lymphocytes responsible for Ig and cytokine production.

### 3.7. WJ-HJ Supplementation Enhanced Proliferation and Cytotoxicity of Splenocytes

**Figure 7:**
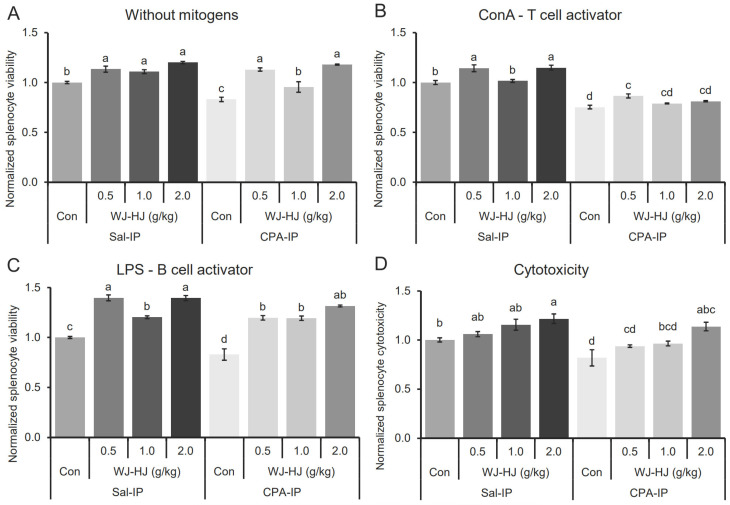
Enhanced viability and cytotoxicity of splenocytes with WJ-HJ supplementation. (**A**) Significant differences were observed in splenocyte viability between the Sal-IP and CPA-IP groups without mitogen treatment. The reduced viability in the Con subgroup of the CPA-IP group was restored in all three WJ-HJ subgroups. Additionally, splenocyte viability was significantly increased in the three WJ-HJ subgroups of the Sal-IP group compared to the Con subgroup. (**B**) When T-cells were activated by ConA, there were significant differences in splenocyte viability between the Sal-IP and CPA-IP groups. Reduced splenocyte viability in the CPA-IP group was restored by WJ-HJ supplementation, while splenocyte viability was enhanced in the three WJ-HJ subgroups of the Sal-IP group compared to the Con subgroup. (**C**) Upon B-cell activation by LPS, splenocyte viability showed significant differences between the Sal-IP and CPA-IP groups. WJ-HJ supplementation restored reduced splenocyte viability in the CPA-IP group and enhanced viability in the Sal-IP group. (**D**) Splenocyte cytotoxicity against cancer cells also differed significantly between the Sal-IP and CPA-IP groups. Similar to the viability results, WJ-HJ supplementation restored reduced splenocyte cytotoxicity in the CPA-IP group and dose-dependently increased cytotoxicity in the Sal-IP group. Different letters above error bars indicate significant differences at *p* < 0.05.

To further investigate the mechanism by which WJ-HJ may counteract CPA-IP-induced immunosuppression, the proliferation and cytotoxicity of splenocytes isolated from the spleens of the Sal-IP and CPA-IP groups were examined (Figure 7). In the absence of mitogens, splenocyte viability in the Con subgroup of the CPA-IP group was significantly lower than that in other subgroups within both the Sal-IP and CPA-IP groups (Figure 7A, F_(7,24)_ = 25.117, *p* = 1.4 × 10^−9^). WJ-HJ supplementation increased splenocyte viability in the Sal-IP and CPA-IP groups.

When concanavalin A (ConA) was used as a mitogen to activate T-lymphocytes, significant differences in splenocyte viability were observed among the Sal-IP and CPA-IP groups (Figure 7B, F_(7,24)_ = 61.429, *p* = 8.8 × 10^−14^). The reduced splenocyte viability in the Con subgroup of the CPA-IP group was restored in the 0.5 g WJ-HJ subgroup. Additionally, splenocyte viabilities in the 0.5 g and 2.0 g WJ-HJ subgroups of the Sal-IP group were significantly higher than in the Con subgroup of the Sal-IP group.

When LPS was used as a mitogen for activating B-cells, there were significant differences in splenocyte viabilities among the Sal-IP and CPA-IP groups (Figure 7C, F_(7,24)_ = 49.732, *p* = 9.3 × 10^−13^). The reduced splenocyte viability in the Con subgroup of the CPA-IP group was completely restored in all three WJ-HJ subgroups. Furthermore, splenocyte viabilities in the three WJ-HJ subgroups within the Sal-IP group were significantly higher than in the Con subgroup in the Sal-IP group.

The cytotoxicity of splenocytes against cancer cells in the Sal-IP and CPA-IP groups was also evaluated (Figure 7D). There were significant differences in splenocyte cytotoxicity across the Sal-IP and CPA-IP groups (F_(7,31)_ = 8.567, *p* = 2.9 × 10^−5^). Cytotoxicity in the Con subgroup of the CPA-IP group was the lowest and was restored in the 2.0 g WJ-HJ subgroup. In the Sal-IP group, splenocyte cytotoxicity increased dose-dependently with WJ-HJ.

These findings indicated that reduced splenocyte viability and cytotoxicity in CPA-IP-induced immunosuppression mice could be restored by WJ-HJ supplementation.

## 4. Discussion

With advancements in biomedical science and medical technology, human life expectancy has significantly increased, with projections suggesting that women in developed countries may surpass 90 years by 2030 [36]. However, the healthspan—the disease-free period of life—lags nearly a decade behind life expectancy, leaving many elderly individuals vulnerable to multiple diseases. This growing aging population, prone to health issues, is driving up healthcare costs and posing challenges to societal stability [37]. Extending healthspan can be achieved through proper nutrition, regular exercise, routine health checkups, and healthy lifestyle choices [37,38]. Studies highlight the potential of foods or drugs to enhance healthspan, with recent research emphasizing the critical role of mitochondrial activity in cellular energy, metabolic regulation, and disease prevention. Mitochondrial dysfunction is closely associated with cancer and neurological, metabolic, cardiovascular, and autoimmune diseases [39,40].

Recent studies also suggest that the immune responses of NK cells [28] and macrophages [41], which play a central role in innate immunity, are activated, and immunity is enhanced when their mitochondrial functions are improved. Therefore, the most important contribution of this study is the discovery that the proliferation and immune surveillance functions of human NK92-MI cells are enhanced by WJ-SCE through mitochondrial function improvement (Figure 1, Figure 2 and Figure 3) and the increased expression of NK cell markers, cytotoxic enzymes, and specific receptors (Figure 4).

The capacity of NK cells to detect and eliminate cancer cells arising from environmental factors or genetic mutations is vital for maintaining health. Individual differences in sensitivity to carcinogens may be partially due to variations in NK cell immune surveillance activity [42]. NK cells can rapidly eliminate nearby cancer cells, inspiring the development of therapy [43]. Clinical studies have shown that separating autologous NK cells, proliferating and activating them ex vivo, and then infusing them back into the patient can help eradicate certain solid tumors [44]. NK cell immunotherapies using various allogeneic sources are also under development, including the NK92-MI cells used in this study [45]. Thus, another significant contribution of this study is demonstrating a method to enhance the function of NK92-MI cells without the use of costly cytokines.

To confirm the immune-enhancing effects of WJ-HJ in vivo, we used a CPA-IP animal model. CPA suppresses the production and proliferation of various immune cells, including NK cells, in mice [46,47], similar to its effects in humans [48]. In the WJ-HJ groups, CPA-IP-induced changes in BW and spleen/liver indices were recovered, although the thymus index did not (Figure 5). This may be because the thymus is a primary lymphoid organ where T-lymphocytes proliferate and mature. In mice, thymus cell numbers begin to sharply decline after 4 weeks, and the organ’s size decreases with age [49]. Since CPA-IP causes T-lymphocyte depletion in the thymus, it is unlikely that the size of the thymus would recover after this point. By contrast, the spleen is a secondary lymphoid organ, where various immune cells such as NK cells, macrophages, T-lymphocytes, and B-lymphocytes, generated in the primary lymphoid organs, migrate to mature and then move to the blood or other tissues [50]. Therefore, the reduction in spleen weights induced by CPA-IP could be recovered in mice supplemented with WJ-HJ (Figure 5). Supporting these findings, the splenocyte viability study demonstrated increased viability in both the CPA-IP and Sal-IP groups, irrespective of mitogen treatment (Figure 7). With ConA, known as a T-lymphocyte activator [51], the viability of splenocytes increased in some of the treatment groups. However, a more pronounced increase was observed when B-lymphocyte activator LPS [52] was used. These results suggest that WJ-HJ has a more substantial effect on B-lymphocytes than T-lymphocytes. This observation may also explain the increased Ig levels of in the blood of the WJ-HJ groups (Figure 6).

Another critical immunomodulatory function of WJ-HJ discovered in this study is its ability to help maintain cytokine homeostasis. CPA-IP is known to alter cytokine levels in mice [53,54]. In this study, IL-6, IL-1β, and TNF-α levels in the CPA-IP group decreased, but these levels were restored with WJ-HJ supplementation (Figure 6D–F), indicating a recovery of immune cell function responsible for secreting these cytokines.

**Figure 8:**
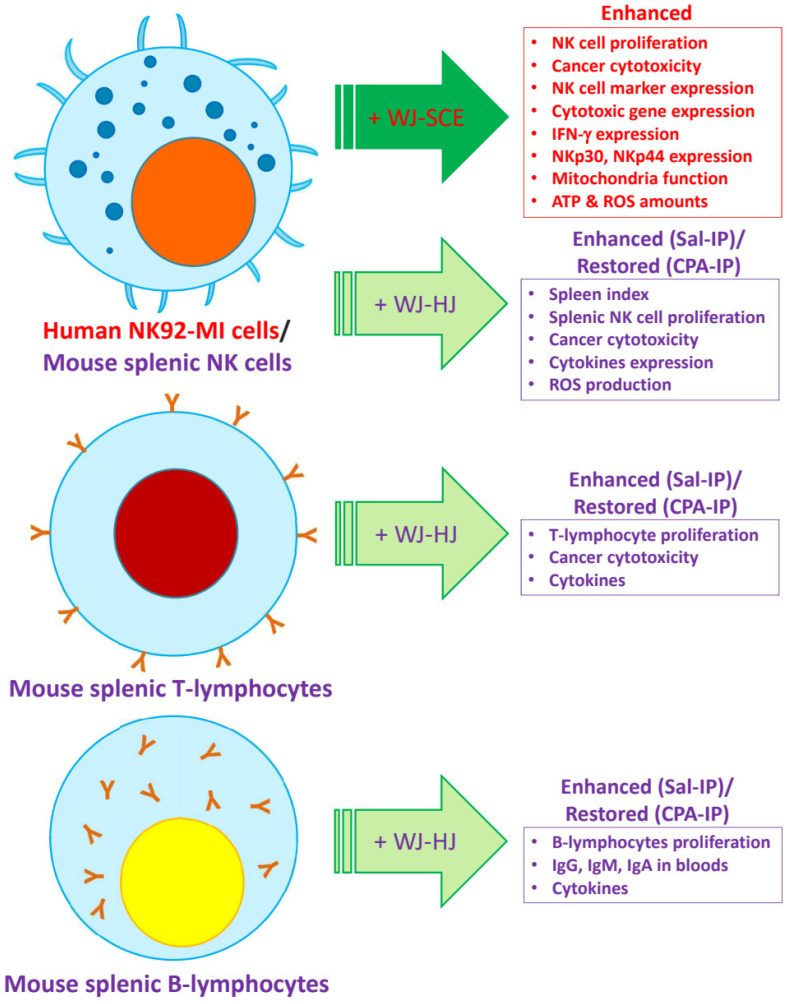
The immune-enhancing effects of supplementation with WJ-SCE in NK92-MI cells and WJ-HJ in the CPA-IP-induced immunosuppressed mouse model. WJ-SCE significantly enhanced proliferation, cancer cytotoxicity, NK cell marker expression, cytotoxic gene expression, IFN-γ expression, and NKp30/NKp44 expression in human NK92-MI cells. In the CPA-IP-induced immunosuppressed mouse model supplemented with WJ-HJ, spleen indices were restored, and splenic NK cells exhibited enhanced proliferation, increased cancer cytotoxicity, elevated cytokine expression, and higher ROS production. Splenic T-lymphocytes from the WJ-HJ-supplemented Sal-IP groups demonstrated enhanced proliferation and cancer cytotoxicity, while T-lymphocytes from the CPA-IP immunosuppressed groups showed restored functionality. Similarly, splenic B-lymphocytes from the WJ-HJ-supplemented Sal-IP groups displayed enhanced proliferation and increased levels of immunoglobulins and cytokines, while those from the CPA-IP immunosuppressed groups exhibited restored B-cell functionality.

WJ-SCE significantly enhanced the proliferation, immunomodulatory, and immune surveillance functions of NK92-MI cells by improving mitochondrial function and upregulating NK cell markers, cytotoxic enzymes, and specific receptors. In vivo, WJ-HJ supplementation effectively mitigated CPA-IP-induced immunosuppression by restoring body and immune organ weights and blood immunoglobulin and cytokine levels and enhancing splenocyte proliferation and cytotoxicity (Figure 8).

## 5. Conclusions

Our findings suggest that WJ-SCE has potential as a therapeutic agent to enhance NK cell cytotoxicity in cancer immunotherapies, while WJ-HJ could be developed into a functional health food aimed at improving immunity and preventing immune-related disorders. Future clinical studies are necessary to validate these immune-enhancing effects in humans, particularly for applications in cancer prevention, recovery from immunosuppression, and disease management.

## 6. Patents

A composition containing HongJam extracts has the effect of promoting the proliferation of Natural killer cells and promoting their anti-cancer effects; Patent Application No: 10-2023-0169360 (Republic of Korea Patent Office, 29 November 2023).

## Data Availability

The original contributions presented in this study are included in the article. Further inquiries can be directed to the corresponding author.

## Supplementary Materials

The following supporting information can be downloaded at: https://www.mdpi.com/article/10.3390/nu17030417/s1, Figure S1: Experimental outline of the CPA-IP-induced immunosuppression mouse model and the doses of supplemented WJ-HJ; Figure S2: A study on changes in body weight of experimental animals supplemented with various feeds in a control group and a CPA-induced immunosuppressed mouse model; Table S1: The list of primers, their DNA sequences, and conditions for RT-qPCR analyses.

## Abbreviations

The following abbreviations are used in this manuscript:

HJ	HongJam
NK92-MI	human interleukin-2-independent Natural Killer 92
CPA-IP	cyclophosphamide-intraperitoneal-injection-induced immunosuppression mouse model
WJ-HJ	White Jade variety mature silkworm HJ
WJ-SCE	WJ-HJ supercritical fluid extract
CPA	cyclophosphamide
NIAS	National Institute of Agricultural Sciences
LDH	lactate dehydrogenase
A490	absorbance at 490 nm
PI	propidium iodide
DPBS	Dulbecco’s phosphate-buffered saline
RT-qPCR	real-time quantitative PCR
MitoCom	Mitochondria complex
MEB	mitochondrial extraction buffer
A600	absorbance at 600 nm
A550	absorbance at 550 nm
Nf	normal mouse feed
Sal-IP	Intraperitoneal injection of saline
BW	body weight
Ig	immunoglobulin
IL	interleukin
ELISA	enzyme-linked immunosorbent assay
ConA	concanavalin A
LPS	lipopolysaccharide
Con	control
NCAM/CD56	neural cell adhesion molecule 1
