# Supplementary material for: Enhanced Immune Functions of In Vitro Human Natural Killer Cells and Splenocytes in Immunosuppressed Mice Supplemented with Mature Silkworm Products
Source: Nutrients. 2025 Jan 23;17(3):417. doi: 10.3390/nu17030417 (PMC11820884; doi:10.3390/nu17030417)
Supplement: Supplementary file 1 [file nutrients-17-00417-s001.zip › nutrients-3417863-supplementary.pdf]

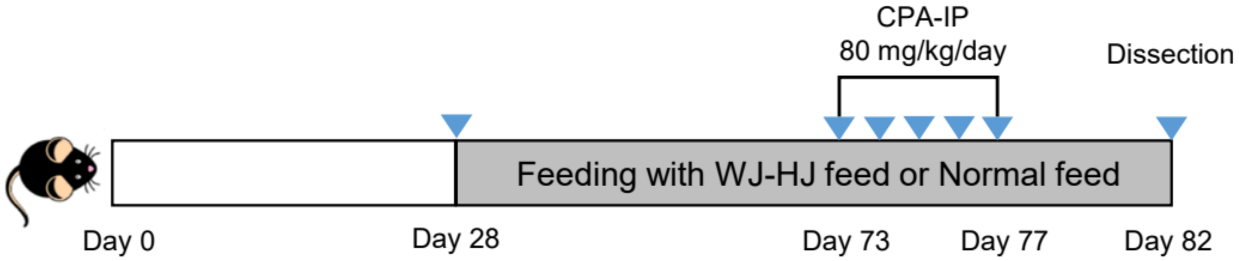

Experimental Subtances		Con Normal Feed (Nf)	WJ-HJ feed		
			0.5 g/kg	1.0 g/kg	2.0 g/kg
IP Injection	Sal	4	4	4	4
	CPA	4	4	4	4

Figure S1. Experimental outline of the CPA-IP-induced immunosuppression mouse model and the doses of supplemented WJ-HJ

Mice were acclimated for one week before starting experiments. To randomly divide 32 experimental animals, each mouse was assigned a unique number from 1 to 32. Using the lottery method, 8 mice per group were alternately selected and assigned to either the Sal-IP or CPA-IP subgroup. For the double-blinded tests, an independent individual administered feeds and intraperitoneal (IP) injections to the mice without knowledge of their content. Additionally, the positions of the mouse cages and the order of injections were rotated daily to eliminate potential confounding effects. After 45 days of feeding the experimental substances, the mice received Sal- or CPA-IP injections (80 mg/kg body weight) from days 73 to 77. The appropriate amounts of WJ-HJ were mixed with standard feed to prepare three types of WJ-HJ feed.

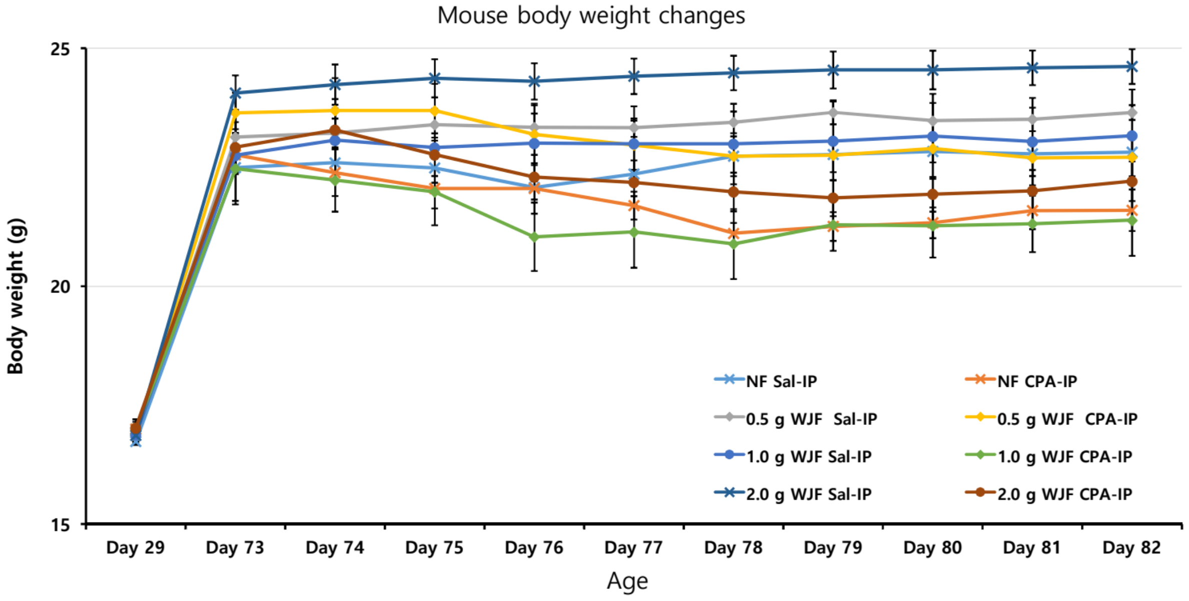

Figure S2. A study on changes in body weight of experimental animals supplemented with various feeds in a control group and a CPA-induced immunosuppressed mouse model.

Significant differences in body weight were observed with aging ($F_{(7, 264)} = 25.68$, P = 1.1 x 10^{-26}). Additionally, there were significant differences in body weight between IPs and feeds ($F_{(7, 264)} = 25.68$, P = 1.8 x 10^{-75}). However, aging did not significantly influence the effects of IPs and feeds on body weight ($F_{(7, 264)} = 0.6375$, P = 0.98).

Table S1. The list of primers, their DNA sequences, and conditions for RT-qPCR analyses

<table>
<tr><th></th><th colspan="2">DNA sequences of oligomers</th><th>PCR conditions</th></tr>
<tr><td rowspan="2">CD56</td><td>F</td><td>5′-TCTGGATGGGCACATGGTG-3'</td><td rowspan="18">95ºC-2 min, 1 cycle;
95ºC-10 sec, 60ºC-15sec, 72ºC-15sec, 40 cycle</td></tr>
<tr><td>R</td><td>5′-TGCTCTTCAGGGTCAGCGA-3'</td></tr>
<tr><td rowspan="2">Granzyme A</td><td>F</td><td>5′-GCGAGGTGACTAACTT-3'</td></tr>
<tr><td>R</td><td>5′-TGACTCTCTCAGAGTATCGGA-3′</td></tr>
<tr><td rowspan="2">Granzyme B</td><td>F</td><td>5′-GAAGTGCGATCTGACTTACG-3'</td></tr>
<tr><td>R</td><td>5′-TTGTTTCGTCCATAGGAGACAA-3'</td></tr>
<tr><td rowspan="2">Perfornin-1</td><td>F</td><td>5′-GCTATCGTTAGTGCTAGTGGAT-3’</td></tr>
<tr><td>R</td><td>5′-ATCTGTCTGATGCGTATCCAAT-3</td></tr>
<tr><td rowspan="2">IFN-γ</td><td>F</td><td>5′-AGCTCTGCATCGTTTTGG GTT-3'</td></tr>
<tr><td>R</td><td>5′-GTCCATATCCGCTACATCTGAA-3'</td></tr>
<tr><td rowspan="2">NKp30</td><td>F</td><td>5′-GCTGGTGGTGGAGAAAGAAC-3′</td></tr>
<tr><td>R</td><td>5′-GGACCTTTCCAGGTCAGACA-3′</td></tr>
<tr><td rowspan="2">NKp44</td><td>F</td><td>5′-TCACAGCCACAGAACTCCAC-3′</td></tr>
<tr><td>R</td><td>5′-CCTGAGCTCCATCATGGTTT-3</td></tr>
<tr><td rowspan="2">NKp46</td><td>F</td><td>5′-TGCCGTCTAGACACTGCAAC-3′</td></tr>
<tr><td>R</td><td>5′-CCAAAACATCGGTATGTCCC-3′</td></tr>
<tr><td rowspan="2">β-actin</td><td>F</td><td>5′-GGCACCCAGCACAATGAAG-3'</td></tr>
<tr><td>R</td><td>5′-GCCGATCCACACGGAGTACT-3'</td></tr>
</table>
